# Supplementary material for: Corona was scary, lockdown was worse: A mixed-methods study of community perceptions on COVID-19 from urban informal settlements of Mumbai
Source: PLoS One. 2022 May 6;17(5):e0268133. doi: 10.1371/journal.pone.0268133 (PMC9075633; doi:10.1371/journal.pone.0268133)
Supplement: S1 File — (DOCX) [file pone.0268133.s001.docx]

**COVID-19 AWARENESS, ATTITUDES AND PRACTICES IN THE COMMUNITY**

**Interview/discussion guide**

**Basic demographics**

| Age |  |
| --- | --- |
| Gender |  |
| Religion |  |
| Marital status |  |
| Employment |  |
| Spouse Employment |  |
| No. of years residing in that community |  |
| Family description |  |

**1: General Questions**

1. How long have you been in this basti? Do you live with your family? Tell me about your family members?
2. Do you work somewhere; can you please tell me what do you do? How is it going these days?

**2: Awareness about transmission, Symptoms of Covid-19, Sources of information, Gendered knowledge**

1. When did you first hear about Corona virus? From where did you hear about it?
2. Current sources of information? (preferred/trusted source)
3. What is the latest news, you have heard about corona?
4. Can you please tell me, what do you know about corona? (ways in which is spread, symptoms, others)
5. Do you think coronavirus affects everyone in the community the same way? Who, according to you does it affected more? In your opinion, does it affect women more or men more (probe for ways)

**3: Current status opinion**

1. Do you think coronavirus is still there in the community? (probe: in your opinion, is it gone? Is there still danger from the disease to you? What are your neighbours saying about this?)

**4: Attitudes towards COVID-19**

1. When you first learned about this infection, what did you feel?
2. When corona cases started increasing in Dharavi, tell me about your experience of that time? How was it for you and your family?
3. How do you feel now compared to march?
4. What do you think people in community feel now?

***Corona stories***

-Did anyone you know get corona in the neighbourhood? Did anyone from your family get? Did you hear any stories? (probe for stories of corona that people have heard- these will give you a hook to ask further questions).

-probe for stigma, experiences of stigma, isolation, fear (if any)

- in the story probe for people’s feelings about how they felt when they encountered corona, in what ways did corona affect women-in these stories. How was this different from the way it affected men? How did the children react? What challenges were faced by people?

5. In your opinion, can corona happen to anyone?

6. Do you feel you and your family are at risk? (what did you feel earlier? What do you feel today?) also ask, why, in your opinion, did * person get corona (where do you think they got it from)

**5: Practices**

1. There are so many things that people say will prevent corona. Could you tell me about some of these ways? (Probe for mask-wearing, handwashing etc)- others.
2. **Mask-wearing**
3. What is your opinion on wearing a mask? Why do you think people wear a mask?
4. Who do you think should wear a mask? Does everyone need to wear one?
5. In your opinion, when is a mask needed? Is it needed by old and young people? Men and women? Do you need it in the house? (to avoid socially desirable answers)- some people told us wearing a mask is uncomfortable, also- we can’t make kids wear it. What do you think? If it is uncomfortable, how do you manage?
6. Some people told us only men need to wear masks?
7. Some told us mask make no difference, so they don’t wear. What do you think?
8. How long do you think mask-wearing should continue? Also, probe for changes in mask-wearing patterns between march and now.
9. Where did you get masks from your family from? How much did it cost?
10. **Hand-washing**
11. What is your opinion on washing hands to prevent corona?
12. Do you think washing hands helps to prevent corona? When do you think one should wash hands?
13. We heard that lack of water makes it challenging to wash hands and sanitize all the time. Could you tell us about you water situation and how you managed?
14. Some people felt that we don’t need to follow washing hands procedures anymore, what do you think about it?
15. **Trust in preventive measures**
16. Do you know of people who washed hands and wore masks- and still got corona?
17. In your opinion, is wearing masks and washing hands enough to prevent corona? What else, in your opinion, needs to be done?
18. Can you describe the precaution your family takes when someone comes home from outside?

**6: Health Systems**

1. During this period, did you/your family require to use any health services?
2. Were you able to access these services? Did you face any challenges?
3. How was your experience? (connect to previous experience of community corona story and ask about health system access)
4. If someone has symptoms like corona, what should they do?
5. During lockdown, were people going to hospitals for any other ailments?
6. How is it now? Do you feel any difference in the way people used health services earlier?

**7: Public services**

1. Probe on services available (food, sanitation, helplines) from the government
2. Probe on surveys done

**8. Challenges**

1. What have been some of your biggest worries/challenges in the last six months?

Some people told us that employment is a problem? What is your opinion on this? (probe for food challenges if any persist, other issues)
