# Supplementary material for: Corona was scary, lockdown was worse: A mixed-methods study of community perceptions on COVID-19 from urban informal settlements of Mumbai
Source: PLoS One. 2022 May 6;17(5):e0268133. doi: 10.1371/journal.pone.0268133 (PMC9075633; doi:10.1371/journal.pone.0268133)
Supplement: S2 File — (DOCX) [file pone.0268133.s002.docx]

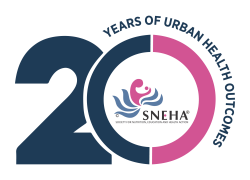


**SNEHA** (**S**ociety for **N**utrition, **E**ducation and **H**ealth **A**ction) I [www.snehamumbai.org](http://www.snehamumbai.org)


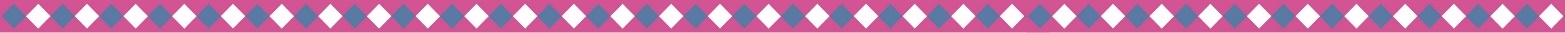


| [Mission](https://www.commcarehq.org/a/sc-baseline/apps/view/5518731be73f08ba4ed8ebe77efcc7e2/module/c798e0a29a2d41bd866eb2b7f7dfe8d1/) Dharavi: Community Survey Questionnaire | | | | | | | | | | | | | | | |
| --- | --- | --- | --- | --- | --- | --- | --- | --- | --- | --- | --- | --- | --- | --- | --- |
| Q -01 | [Program](https://www.commcarehq.org/a/sc-baseline/apps/view/5518731be73f08ba4ed8ebe77efcc7e2/form/3287559ac7ac469381f2aff102c65c62/source/#form/program) Names | | □ Aahar | | | □ Ehsas | | | | | □ PVWC | | | | |
| Q -02 | [Are you willing to share the information?](https://www.commcarehq.org/a/sc-baseline/apps/view/5518731be73f08ba4ed8ebe77efcc7e2/form/3287559ac7ac469381f2aff102c65c62/source/#form/consent) | | | | | | | | | | | | | | |
|  | - - Yes   - No   - Migrated to native place | | | | - - Phone not available   - Shifted residence from area   - Other (Specify) …………………………………………………. | | | | | | | | | | |
| Q - 03 | [Select Area from Dharavi](https://www.commcarehq.org/a/sc-baseline/apps/view/5518731be73f08ba4ed8ebe77efcc7e2/form/3287559ac7ac469381f2aff102c65c62/source/#form/aahar_dharavi) | | | | | | | | | | | | | | |
|  | - - Kumbharwada   - Shed   - Pila Bungalow   - Shastri Nagar   - Naik Nagar   - 90 ft road   - Rajiv Ghandhi Nagar   - AKJ Nagar | - - Abubakar chawl   - Azad nagar   - Bhagat Singh Nagar   - Bhim nagar   - Dhareshwar krida mandal   - Ganesh Vikas mandal   - Indira Nagar   - Kamla Nagar | | | | | - - Kamla nehru nagar   - Khada hanuman chauk   - Kunchikurve nagar   - Matunga Labour camp   - Mahatma Ghandhi nagar   - Mukund nagar   - Muslim nagar | | | | | | - - Ramabai nagar   - Sai baba nagar   - Siddharta nagar   - Sidhartha chawl   - Social nagar   - Subhash nagar   - Other | | |
| Q - 04 | Write the name of the beneficiary | | | ………………………………………………………………………………………………………… | | | | | | | | | | | |
| Q - 05 | Age | | | □□ | | | | | | | | | | | |
| Q - 06 | [Sex](https://www.commcarehq.org/a/sc-baseline/apps/view/5518731be73f08ba4ed8ebe77efcc7e2/form/3287559ac7ac469381f2aff102c65c62/source/#form/sex) | | | □ Male | | | | | □ Female | | | | | □ Other | |
| Q - 07 | [How many people live in your household, including yourself?](https://www.commcarehq.org/a/sc-baseline/apps/view/5518731be73f08ba4ed8ebe77efcc7e2/form/3287559ac7ac469381f2aff102c65c62/source/#form/hh_members) | | | □□ | | | | | | | | | | | |
| Information and channels | | | | | | | | | | | | | | | |
| Q - 08 | [Are you aware of Corona virus/COVID-19?](https://www.commcarehq.org/a/sc-baseline/apps/view/5518731be73f08ba4ed8ebe77efcc7e2/form/3287559ac7ac469381f2aff102c65c62/source/#form/covid19aware) | | | | | | | - Yes | | | | - No | | | |
| Q - 09 | [Where did you hear about the new coronavirus from?](https://www.commcarehq.org/a/sc-baseline/apps/view/5518731be73f08ba4ed8ebe77efcc7e2/form/3287559ac7ac469381f2aff102c65c62/source/#form/hear) | | | | | | | | | | | | | | |
|  | - - Did not hear about the coronavirus   - Radio   - TV   - Local cable network   - Covid Yoddhas   - Posters/Print advertisements   - Religious places   - Awareness sessions   - Group meetings   - Events/campaigns   - Public Announcement with Megaphone   - WhatsApp | | | | | - - Social media (other than WhatsApp. Like Facebook, TikTok, Twitter, etc.)   - Family/friends   - Community health workers (ICDS, MCGM)   - Pharmacy   - Traditional healers   - Community leaders   - Religious leaders   - SNEHA   - Other NGOs   - Google/internet   - Other ([Specify)](https://www.commcarehq.org/a/sc-baseline/apps/view/5518731be73f08ba4ed8ebe77efcc7e2/form/3287559ac7ac469381f2aff102c65c62/source/#form/corona_hear_other) ……………………………………………… | | | | | | | | | |
| Q - 10 | Of these sources which one you trust the most? Include list of options selected | | | | | | | | | | | | | | |
|  | - - Did not hear about the coronavirus   - Radio   - TV   - Local cable network   - Covid Yoddhas   - Posters/Print advertisements   - Religious places   - Awareness sessions   - Group meetings   - Events/campaigns   - Public Announcement with Megaphone   WhatsApp | | | | | - - Social media (other than WhatsApp. Like Facebook, TikTok, Twitter, etc.)   - Family/friends   - Community health workers (ICDS, MCGM)   - Pharmacy   - Traditional healers   - Community leaders   - Religious leaders   - SNEHA   - Other NGOs   - Google/internet   - Other ([Specify )](https://www.commcarehq.org/a/sc-baseline/apps/view/5518731be73f08ba4ed8ebe77efcc7e2/form/3287559ac7ac469381f2aff102c65c62/source/#form/corona_hear_other) ……………………………………………… | | | | | | | | | |
| Q - 11 | Do you think that [source] would be a good source for getting useful information about Coronavirus? | | | | | | | | | | | | | | |
|  | Did not hear about the coronavirus | | | | | | | | | □ Yes | | | | | □ No |
|  | Radio | | | | | | | | | □ Yes | | | | | □ No |
|  | TV | | | | | | | | | □ Yes | | | | | □ No |
|  | Local cable network | | | | | | | | | □ Yes | | | | | □ No |
|  | Covid Yoddhas | | | | | | | | | □ Yes | | | | | □ No |
|  | Posters/Print advertisements | | | | | | | | | □ Yes | | | | | □ No |
|  | Religious places | | | | | | | | | □ Yes | | | | | □ No |
|  | Awareness sessions | | | | | | | | | □ Yes | | | | | □ No |
|  | Group meetings | | | | | | | | | □ Yes | | | | | □ No |
|  | Events/campaigns | | | | | | | | | □ Yes | | | | | □ No |
|  | Public Announcement with Megaphone | | | | | | | | | □ Yes | | | | | □ No |
|  | WhatsApp | | | | | | | | | □ Yes | | | | | □ No |
|  | Social media (other than WhatsApp. Like Facebook, TikTok, Twitter, etc.) | | | | | | | | | □ Yes | | | | | □ No |
|  | Family/friends | | | | | | | | | □ Yes | | | | | □ No |
|  | Community health workers (ICDS, MCGM) | | | | | | | | | □ Yes | | | | | □ No |
|  | Pharmacy | | | | | | | | | □ Yes | | | | | □ No |
|  | Traditional healers | | | | | | | | | □ Yes | | | | | □ No |
|  | Community leaders | | | | | | | | | □ Yes | | | | | □ No |
|  | Religious leaders | | | | | | | | | □ Yes | | | | | □ No |
|  | SNEHA | | | | | | | | | □ Yes | | | | | □ No |
|  | Other NGOs | | | | | | | | | □ Yes | | | | | □ No |
|  | Google/internet | | | | | | | | | □ Yes | | | | | □ No |
|  | Other | | | | | | | | | □ Yes | | | | | □ No |

| Program recall: awareness about Covid-19(Symptoms, transmission, high-risk groups, prevention) | | | | | |
| --- | --- | --- | --- | --- | --- |
| Q - 12 | [How does the coronavirus spread?](https://www.commcarehq.org/a/sc-baseline/apps/view/5518731be73f08ba4ed8ebe77efcc7e2/form/0a3a0a0519ea46d88d9d4a1fe7659e7f/source/#form/corona_spread) | | | | |
|  | - - Blood transfusion   - Droplets from infected people   - Airborne   - Direct contact with infected people. | - - Touching contaminated objects/surfaces   - Sexual intercourse contact   - Contact with contaminated animals   - Mosquito bites | | | - - Eating contaminated food   - Drinking unclean water   - Don't know   - Other([Specify](https://www.commcarehq.org/a/sc-baseline/apps/view/5518731be73f08ba4ed8ebe77efcc7e2/form/3287559ac7ac469381f2aff102c65c62/source/#form/symptoms_others))   ………………………………………………..………. |
| Q - 13 | [What are the main symptoms of Coronavirus/COVID-19?](https://www.commcarehq.org/a/sc-baseline/apps/view/5518731be73f08ba4ed8ebe77efcc7e2/form/3287559ac7ac469381f2aff102c65c62/source/#form/symptoms) | | | | |
|  | - - Don't know   - Fever or chills   - Dry cough.   - Tiredness./Fatigue   - Shortness of breath or difficulty breathing   - Muscle or body aches | - - Sore throat.   - Headache   - Loss of taste or smell   - Congestion or runny nose   - Nausea or vomiting   - Diarrhea | | | - - Conjunctivitis.     - A rash on skin, or discolouration of fingers or toes.     - Chest pain or pressure.   - Loss of speech or movement   - Other([Specify](https://www.commcarehq.org/a/sc-baseline/apps/view/5518731be73f08ba4ed8ebe77efcc7e2/form/3287559ac7ac469381f2aff102c65c62/source/#form/symptoms_others))   ………………………………………………..………. |
| Q - 14 | [Do you know how to prevent COVID-19?](https://www.commcarehq.org/a/sc-baseline/apps/view/5518731be73f08ba4ed8ebe77efcc7e2/form/0a3a0a0519ea46d88d9d4a1fe7659e7f/source/#form/prevention) | | | | |
|  | - - Sleep under the mosquito net   - Wash your hands regularly using sanitizer or soap and water   - Drink only treated water/ filtered/boiled water   - Cover your mouth and nose when coughing or sneezing   - Avoid close contact with anyone who has a fever and cough   - Eliminate stagnant water   - Cook meat and eggs well | | | - - Avoid unprotected direct contact with live animals and surfaces in contact with animal   - Social distancing/ Staying indoors   - Wearing face masks   - Use herbal/ayurvedic/ home remedies   - Don't know   - Other([Specify](https://www.commcarehq.org/a/sc-baseline/apps/view/5518731be73f08ba4ed8ebe77efcc7e2/form/3287559ac7ac469381f2aff102c65c62/source/#form/symptoms_others))   ………………………………………………………………………..………. | |
| Perceived threat | | | | | |
| Q - 15 | Do you think your chance of getting infected with Coronavirus is | | | | |
|  | - - Low,   - Medium,   - High | | | - - No risk at all   - Already had COVID-19   - Don’t Know | |
| Q - 16 | Why do you think you are not at high risk? | | | | |
|  | - - I'm young   - God protects me   - Hot weather and Climate   - COVID-19 is not in neighbourhood   - I haven’t travelled   - Don’t know anyone with Covid 19   - I have been staying at home | | - - Have been adhering to government guidelines   - Practice social distancing/staying 1-2 meters apart/not shaking hands/not interacting with people   - Have been washing hands/using sanitizer   - Have been wearing a face mask   - I am healthy   - Other ([Specify](https://www.commcarehq.org/a/sc-baseline/apps/view/5518731be73f08ba4ed8ebe77efcc7e2/form/3287559ac7ac469381f2aff102c65c62/source/#form/symptoms_others))……………………………………………………   - Don't know/no response | | |

| Healthy/protective behaviour | | |
| --- | --- | --- |
| Q - 17 | Are you taking any measures to prevent Coronavirus in past one week ? | |
|  | - - Not doing anything   - Wash hands regularly using alcohol sanitizer or soap and water   - Wear masks if going outdoors   - Social distancing/ Staying indoors | - - Using homeopathic remedies   - Disinfecting surfaces.   - Eating garlic, ginger, lemon   - Other([Specify](https://www.commcarehq.org/a/sc-baseline/apps/view/5518731be73f08ba4ed8ebe77efcc7e2/form/3287559ac7ac469381f2aff102c65c62/source/#form/symptoms_others))   ………………………………………………………………………..………. |
| Q - 18 | [You may be aware of the importance of sanitation and hygiene to prevent Coronavirus. What kind of toilet facility do members of your household usually use?](https://www.commcarehq.org/a/sc-baseline/apps/view/5518731be73f08ba4ed8ebe77efcc7e2/form/3287559ac7ac469381f2aff102c65c62/source/#form/toilet_facility) | |
|  | - - Flush/pour flush toilet in the community   - Private toilet inside home | - - On field or road   - Other ([Specify](https://www.commcarehq.org/a/sc-baseline/apps/view/5518731be73f08ba4ed8ebe77efcc7e2/form/3287559ac7ac469381f2aff102c65c62/source/#form/symptoms_others)) ……………………………………………………. |
| Q - 19 | Can you tell me when are the times that you washed your hands with soap and water or used hand sanitizer yesterday? | |
|  | - - Before preparing food   - Before eating   - After using the toilet   - After changing a baby's faeces   - After coming home from a public place   - After coughing/sneezing | - - Before or after caring for a sick or vulnerable person   - Before leaving the house   - After using the phone   - Before entering a shop/office   - After entering a shop/office - Other ([Specify](https://www.commcarehq.org/a/sc-baseline/apps/view/5518731be73f08ba4ed8ebe77efcc7e2/form/3287559ac7ac469381f2aff102c65c62/source/#form/symptoms_others)) …………………………………………………………………………. |
| Q - 20 | In past one week, have you worn a mask when leaving home? | |
|  | - - Always   - Often | - - Rarely   - Never |
| Q - 21 | Where do you think you should wear a mask? | |
|  | - - Everywhere outside my house   - When walking on the street   - When on public transport | - - In supermarkets/markets/shops   - At work   - Other ([Specify](https://www.commcarehq.org/a/sc-baseline/apps/view/5518731be73f08ba4ed8ebe77efcc7e2/form/3287559ac7ac469381f2aff102c65c62/source/#form/symptoms_others)) …………………………………………………………………………. |
| Q - 22 | In past one week, what kind of mask are you using? | |
|  | - - Reusable cloth masks   - Disposable medical mask | - - Medical surgical mask (N-95)   - Other |
| Q - 23 | In past one week, how often did you wash your reusable cloth masks? | |
|  | - - After every use   - After 2-3 use | - - Rarely   - Never |
| Q - 24 | In past one week, how did you wash your reusable cloth masks? | |
|  | - - With soap and water   - With soap and hot water | - - With water only   - Others |
| Q - 25 | With whom do you share your mask? | |
|  | - - I do not share my mask   - Household members   - Non-household members | - - Other (specify)   - Don't know/refuse to answer |
| Q - 26 | [Are you aware of any Helpline numbers for providing medical/non-medical services?](https://www.commcarehq.org/a/sc-baseline/apps/view/5518731be73f08ba4ed8ebe77efcc7e2/form/3287559ac7ac469381f2aff102c65c62/source/#form/helplineaware) | - - Yes   - No |
| Q - 27 | [If yes, then which of the following are helplines available for?](https://www.commcarehq.org/a/sc-baseline/apps/view/5518731be73f08ba4ed8ebe77efcc7e2/form/3287559ac7ac469381f2aff102c65c62/source/#form/helplinetype) | |
|  | - - Don’t know   - Testing for COVID-19   - Food distribution systems   - Mental health and counselling | - - Child sexual abuse   - Violence against women   - Other |
| Q - 28 | [Have you used it?](https://www.commcarehq.org/a/sc-baseline/apps/view/5518731be73f08ba4ed8ebe77efcc7e2/form/3287559ac7ac469381f2aff102c65c62/source/#form/helplineuse) | - - Yes   - No   [If yes, which one?](https://www.commcarehq.org/a/sc-baseline/apps/view/5518731be73f08ba4ed8ebe77efcc7e2/form/3287559ac7ac469381f2aff102c65c62/source/#form/helplineothertext) …………………………………………………. |
| Q - 29 | If yes, are you satisfied with the response? | - - Yes   - No |
| Perceived efficacy | | |
| Q - 30 | How effective do you think your actions are at reducing the risk of you being infected with coronavirus? | |
|  | - - Very effective   - Somewhat effective | - - A little effective   - Not effective at all |
|  | Others | |
| Q - 31 | In the past 4 weeks, have you/any member of your family been screened Covid-19 symptoms by | |
|  | - - Not screened   - By Covid Yoddhas   - By CHV   - By ANM | - - By AWW   - By Sneha Staff   - By other NGOs   - Others |
| Q - 32 | [What kind of information do you need the most?](https://www.commcarehq.org/a/sc-baseline/apps/view/5518731be73f08ba4ed8ebe77efcc7e2/form/3287559ac7ac469381f2aff102c65c62/source/#form/infoneed) | |
|  | - - Don't want any more information   - How I can protect myself and my family against the Coronavirus?   - How I can access health services if I have Coronavirus   - How I can take care of a person who belongs to a risk group (older people, people with underlying medical conditions such as cardiovascular disease, diabetes, chronic respiratory disease, and cancer)   - How I can best take care of my children’s school education?   - How I will be impacted economically by the pandemic?   - How to maintain my mental health during isolation?   - Information regarding travelling back to native place   - Information about availability of ration/groceries   - Other ([Specify)](https://www.commcarehq.org/a/sc-baseline/apps/view/5518731be73f08ba4ed8ebe77efcc7e2/form/3287559ac7ac469381f2aff102c65c62/source/#form/info_needed_other) …………………………………….………………………….………………………….…………………………. | |
| Q - 33 | [Are you aware of the following activities by the local administration in your area?](https://www.commcarehq.org/a/sc-baseline/apps/view/5518731be73f08ba4ed8ebe77efcc7e2/form/3287559ac7ac469381f2aff102c65c62/source/#form/act) | |
|  | - - Conducting a door to door survey for screening people for Coronavirus.   - Testing suspected cases of Coronavirus and their contacts.   - Quarantine infected patients and their contacts   - Testing senior citizens or high-risk people for Coronavirus/COVID-19   - Distribution of essentials like groceries and medicines.   - Communication about prevention, quarantine, use of masks   - Disinfection of the area/building after a positive case has been found   - Disinfection of public toilets.   - Guarding of containment area by the police.   - Not aware of any of the above. | |
| Q - 34 | [Crises often involve fears and worries. What are the following that you worry about at the moment during this present crisis?](https://www.commcarehq.org/a/sc-baseline/apps/view/5518731be73f08ba4ed8ebe77efcc7e2/form/3287559ac7ac469381f2aff102c65c62/source/#form/crisis) | |
|  | - - Loosing someone I love   - Personal mental health.   - Personal physical health   - Health of your loved ones   - Restricted liberty of movement.   - Restricted access to food supplies | - - Becoming unemployed   - Not being able to pay bills   - Not able to visit people who depend on you   - Health systems being overloaded   - Other Specify……………………………………………………. |
| Q - 35 | [In the past weeks, from where did you get your supply/groceries from?](https://www.commcarehq.org/a/sc-baseline/apps/view/5518731be73f08ba4ed8ebe77efcc7e2/form/3287559ac7ac469381f2aff102c65c62/source/#form/supplies) | |
|  | - Purchased from the grocery store - Got if for free from the grocery store - Got it from SNEHA - Got it from other NGOs/ community leaders/ religious leaders - Had stored food for emergency - Received cooked food - Other Specify………………………………………………………………………………………. | |
| Q - 36 | [In the past 4 weeks, was there ever no food to eat of any kind in your household because of lack of resources to get food?](https://www.commcarehq.org/a/sc-baseline/apps/view/5518731be73f08ba4ed8ebe77efcc7e2/form/3287559ac7ac469381f2aff102c65c62/source/#form/nofood) | - - Yes - No |
| Q - 37 | [How often did this happen in the past weeks?](https://www.commcarehq.org/a/sc-baseline/apps/view/5518731be73f08ba4ed8ebe77efcc7e2/form/3287559ac7ac469381f2aff102c65c62/source/#form/no_food_often) | - - Rarely (once or twice)   - Sometimes (three to ten times)   - Often (more than ten times) |
